# Osteoid Osteoma Presenting as a Painful Solitary Skull Lesion: A Case Report

**Published:** 2014-04

**Authors:** Mohammad Saeed Ahmadi, Mohammad Ahmadi, Arash Dehghan

**Affiliations:** 1 *Department of Otorhinolaryngology, Besat Hospital, Hamadan University of Medical Sciences, Hamedan, Iran.*; 2 *Department of Pathology, Besat Hospital, Hamadan University of Medical Sciences, Hamedan, Iran.*

**Keywords:** Osteoma, Osteoid, Temporal Bone, Skull

## Abstract

**Introduction:**

Osteomas are asymptomatic and rare slow growing bony tumors in temporal bone, and should be taken into account in differential diagnosis of the osteolytic solitary skull lesions. Sometimes is associated with pain and functional loss. Only a few cases have been reported.

**Case Report:**

We describe a case of an osteoid osteoma of the temporal bone (mastoid) in a 31-year-old woman presenting as painful solitary tumor of calvarium and its management. The resection of whole bony tumor was performed using the retroauricular approach. Pathologic evaluation revealed the osteoid osteoma.

**Conclusion:**

Although osteoid osteoma of the temporal bone is rare, it should be taken into account in differential diagnosis of the osteolytic skull lesions. Treatment is indicated for symptomatic osteomas or cosmetic reasons.

## Introduction

Osteoid osteoma is a benign skeletal disorder, and new bone forming tumors located within bones or developing on them. They are often asymptomatic, and are incidentally found on radiological investing- ations. Osteomas are frequently found in the frontal-ethmoid region (1,2). In the temporal bone, the external auditory canal is the predominant location, rarely present in the mastoid, the squamous portion of the temporal bone, inner ear canal and middle ear.3 CT is more accurate than MRI. On CT scans, osteoid osteoma appears as a circumscribed annular lesion with a double-attenuating sign. (). Magnetic resonance image (MRI) reveals typically low signal intensity on T1- and T2-weighted images with contrast enhancement (). Here, we report a rare case of osteoid osteoma presenting as a painful solitary skull lesion.

## Case Report

**Fig1:**
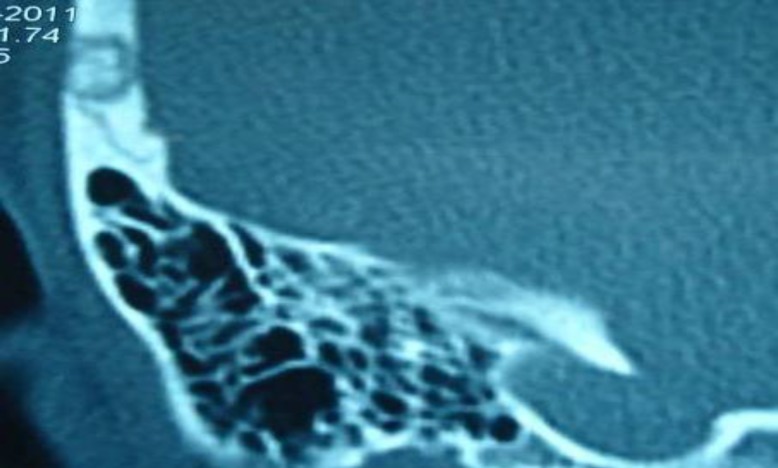
CT Scans Revealing an Osteolytic Lesion With Erosion of the Inner and Outer Skull Tables, and Expansion of the Diploic Space in the Right Temporal Area

This 31-year-old woman presented with a painful scalp lesion on the left mastoid, 2 cm in diameter. She had no specific medical history, and her general condition was good. She experienced severe headache of abrupt-onset 1 month ago, and noticed a newly developed scalp tenderness on the left mastoid region. Findings from physical examination were normal except for a tender scalp lesion on the left temporal region. She was neurologically intact. Routine laboratory data, including serum calcium, phosphorus, and alkaline phosphatase, were within the normal limits. Brain computed tomography scans showed approximately 2.5 cm sized, lobulated, osteolytic lesion over the right temporal (mastoid) bone (Fig.1). 

**Fig2:**
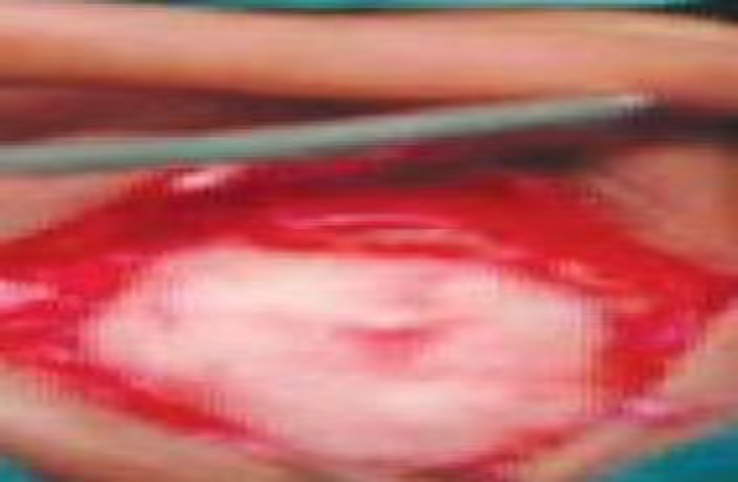
Osteoid Osteoma Intraoperative

During the operation, an reddish lesion was attached to the bone, and had completely eroded the outer and inner tables of skull. At the margins of the lesion, the skull was thickened and gritty in an area of 1 to 1.5 mm around the lesion (Fig.2). En-bloc resection of the bony lesion including surrounding healthy bone enough to clear resection margin was performed. The cranial defect did not repair. After the operation, no neurological deficit was found, and a good cosmetic result was achieved.

**Fig 3:**
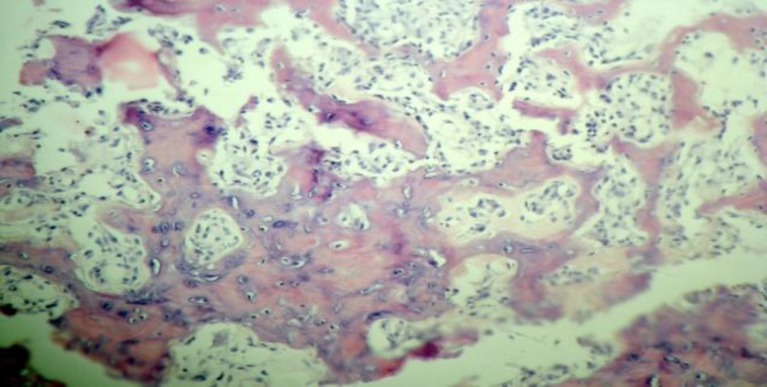
The Tumor Is Composed of Interweaving Trabeculae of Osteoid and Bone Tined by Osteoblasts and a Few Osteoclasts With a Loose Stromal Vascular Connective Tissue Background

Histopathological examination confirmed an osteoid osteoma (Fig.3). She was regularly followed for 10 months without any signs of regrowth. 

## Discussion

Osteoma is a slow growing benign mesenchymal osteoblastic tumor formed by mature bone tissue. Osteoma is prone to grow from the outer table of the cranium, jaw and paranasal sinuses (1). Excluding lesions of the external auditory canal osteomas of the temporal bone are definite rare occurrences, the commonest site being the squama and the mastoid. Generally, osteomas of the temporal bone occur in young individuals, and those of the mastoid process are more common in females. Mastoid osteoma (2) is usually single, and grows from the outer table of the mastoid cortex producing an external swelling. Temporal bone osteomas are rare before puberty. It is generally an incidental finding. Its occurrence is of 0.1% to 1% of all benign tumors of the skull (3). The mastoid osteoma etiology includes trauma, previous surgery, radiotherapy, chronic infection, and hormonal factors with dysfunction in the hypophyseal gland (4). The osteomas are reported in all portions of the temporal bone, including squama, mastoid, internal and external auditory meatus, glenoid cavity, middle ear, Eustachian tube, petrous apex, and styloid process (5-8). Viswanatha B reported that the extracanalicular osteomas of the temporal bone locate mainly in the mastoid portion (9). Giant occipital osteomas can cause dizziness requiring surgical excision (10). Osteoma occurrence may be syndromic or nonsyndromic. They may occur as a feature of Gardener’s syndrome, which is characterized by multiple intestinal polyps, epidermoid inclusion cysts, fibromas of the skin and mesentery and osteomas. Osteomas in Gardener’s syndrome have a predilection for membranous bones, and as such the mandible and maxilla are more commonly involved (11). Treatment is indicated for osteomas that are symptomatic or cosmetically unacceptable. Excision or drilling of superficial lesions of the mastoid and squama is a simple procedure. At surgery, since the lesions are always limited to the external cortex, a cleavage plane is always encountered when tumor meets normal bone (13). Their prognosis has been considered to be good in an esthetic and curative point of view when submitted to surgical exeresis. Recurrence is uncommon and malignant transfor- mation has not been reported in the medical literature (14).

## Conclusion

 We reported a rare case of an osteoid osteoma of the temporal bone (mastoid) in a young woman with severe retroauricular pain. Although osteoid osteoma of temporal bone is an infrequent benign tumor, it should be included in the differential diagnosis of an osteolytic temporal lesion. The suspected diagnosis is based on the clinical findings and CT. Surgical removal is indicated when growth of the osteoma causes distressing symptoms or cosmetic issues. The prognosis of the mastoid osteoma may be considered to be good in cosmetic and curative aspects when it is completely excised.
